# The Focus Group Must Go On: Lessons Learned from Conducting Virtual Focus Groups

**DOI:** 10.1093/geroni/igab046.3062

**Published:** 2021-12-17

**Authors:** Ashlee Cordell, Christa Wilk, Silvia Orsulic-Jeras, Sara Powers, Farida Ejaz, Lisbeth Sanders

**Affiliations:** 1 Benjamin Rose Institute on Aging, Cleveland, Ohio, United States; 2 Benjamin Rose Institute, Cleveland, Ohio, United States; 3 LifeBio, Marysville, Ohio, United States

## Abstract

The Covid-19 pandemic has presented a multitude of challenges in conducting research with human subjects. In response, researchers have found creative ways to complete these studies using alternative methods that incorporate social distancing. Fortunately, numerous technologies exist today that allow individuals to connect with one another over short and long distances. The current study describes the development of LifeBio Memory: an app-based product that utilizes artificial intelligence and machine learning to improve an existing life story intervention designed for persons living with dementia (PWD). Seven focus groups (n=35), originally planned in-person, were successfully converted to a virtual setting. Groups were hosted using a Zoom platform, lasted 75-90 minutes (Mean = 85; SD = 5.3), and consisted of participants from 14 different states: One group of community-dwelling PWDs with early-stage dementia (n=5); two groups of current and former users of the original LifeBio program (n=12); and four groups of residential care staff and directors (n=18). Virtual focus group delivery was determined to be an acceptable and feasible alternative to traditional in-person formats. Topics discussed in this poster will include: 1) recruitment procedures, 2) screening protocols, 3) methods for sharing materials, 4) guidance for providing technology support, and 5) communication strategies to increase retention. Further discussion will focus on challenges faced when collecting data in a virtual setting, tips for successful facilitation, advantages to using virtual alternatives, and other lessons learned from the virtual field.

